# Analysis of the costs and reach of transportation services to reduce barriers to opioid use disorder (OUD) treatment: evidence from the Kentucky HEALing Communities Study

**DOI:** 10.1186/s13722-026-00702-1

**Published:** 2026-07-20

**Authors:** Drew D. Speer, Joshua L. Bush, Don Lochana Ekanayake, Julie Nakayima, Michael M. Goetz, Jennifer Miles, Elizabeth Larimore, Hannah K. Knudsen, Sharon L. Walsh, Kathryn E. McCollister

**Affiliations:** 1 Substance Use Priority Research Area, University of Kentucky, 845 Angliana Avenue, Lexington, KY 40508 USA; 2 College of Public Health, University of Kentucky, 111 Washington Avenue, Lexington, KY 40536 USA; 3 Kentucky Injury Prevention Research Center, University of Kentucky, 2365 Harrodsburg Road, Southcreek Building B, Suite B475, Lexington, KY 40504 USA; 4 Department of Public Health Sciences, University of Miami Miller School of Medicine, 1120 NW 14th Street, Suite 1019, Miami, FL 33136 USA; 5 Department of Behavioral Science, University of Kentucky, 845 Angliana Avenue, Lexington, KY 40508 USA; 6 Center on Drug and Alcohol Research, College of Medicine, University of Kentucky, 845 Angliana Avenue, Lexington, KY 40508 USA

**Keywords:** Transportation, Substance use, Economics, OUD policy, Barriers to care

## Abstract

**Background:**

Medications for opioid use disorder (MOUD) have been proven to be the most effective method for treating individuals with opioid use disorder (OUD). Individuals who are retained on methadone and buprenorphine treatment have a decreased risk of mortality when compared to others with OUD. However, many individuals in need of MOUD face transportation barriers that prevent them from having the requisite access to treatment.

**Methods:**

As part of the evidence-based practices (EBPs) implemented throughout the HEALing (Helping to End Addiction Long-term^®^) Communities Study (HCS), strategies to reduce transportation barriers to receiving methadone and buprenorphine at partner organizations were identified and implemented. Multiple strategies were employed including transportation agency contracts, bus passes, rideshare services, leased agency vans, and fuel cards. This study provides a description of the number of unique riders, including demographic and geographic characteristics, and the costs of providing these transportation services across participating counties.

**Results:**

Throughout 2023, we partnered with thirty agencies across eight counties to administer $437,481.69 in transportation support services to 7,923 individuals. These services were distributed primarily to individuals who were white (81.8%; 6,483), female (51.9%; 4,112) and between the ages of 35–54 (59.8%; 4,737). The number of individuals reached and expenses were largest among the five urban counties (5,999; $318,317.15), which averaged $53.06 per person. Within the five urban counties, we worked with 23 agency partners and the average agency costs were $10,993.72. Across the three rural counties, costs averaged $61.94 per person reached (1,924; $119,164.54). Among the seven agency partners across the three rural counties, average agency costs were $9,235.38.

**Conclusions:**

By gaining an understanding of the populations benefiting from these services, breadth of services provided, and the costs required to address this barrier to care, we may more effectively inform decision-makers on the resources needed to link and retain individuals in treatment.

**Trial registration:**

ClinicalTrials.gov, NCT04111939. Registered 30 September 2019, https://www.clinicaltrials.gov/ct2/show/NCT04111939.

## Introduction

The Centers for Disease Control and Prevention reported that drug overdose deaths involving opioids exceeded 80,000 for the third consecutive year in 2023 [1]. Nearly 70% of these overdose deaths involved synthetic opioids, primarily fentanyl [1]. To address drug overdose mortality, in 2021, the U.S. Department of Health & Human Services (DHHS) released an Overdose Prevention Strategy, which aims to support four strategic priorities: primary prevention, harm reduction, evidence-based treatment, and recovery support [2]. Within the evidence-based treatment strategies, two FDA-approved medications for opioid use disorder (MOUD), buprenorphine and methadone, are effective in reducing opioid use and overdose among individuals with opioid use disorder (OUD) [3]. These medications are safe and designed for long-term use and work by normalizing brain chemistry to block the euphoric effects, relieve cravings, and restore normal body functions. Use of these medications may range from months to years and like all medications taken over a long duration, they require regular healthcare visits and, for buprenorphine and naltrexone, access to a pharmacy that stocks these medications [4–6]. Although telehealth emerged during the COVID-19 pandemic as a strategy to maintain access to MOUD treatment, the vast majority of buprenorphine initiations continue to occur in-person, and states vary considerably in their requirements regarding in-person visits for buprenorphine initiation and ongoing treatment [7]. For methadone, although the percentage of patients reporting weekly or more in-person visits decreased during the COVID-19 pandemic, many patients were still required to attend visits in-person [8, 9].

Furthermore, uptake of these life-saving medications is low with estimates suggesting that only around 25% of individuals with OUD receive MOUD [10, 11]. In addition to the low uptake of MOUD, retention in care is also poor and discontinuing treatment increases the risk of relapse and overdose [12]. Studies have shown that engaging in treatment longer lowers the odds of emergency department visits and inpatient hospital visits and is associated with reductions in morbidity and mortality [13, 14]. However, prior research has estimated that retention rates vary from just 30–50% in most settings, with a variety of factors contributing to this poor retention [14].

Access to transportation is one of the most widely cited barriers to engaging in healthcare, and lack of transportation is a key barrier to MOUD retention [15–23]. People with chronic diseases, such as OUD, are disproportionately impacted by transportation barriers [24–27]. Individuals with OUD are often required to visit opioid treatment providers daily or near daily, or physician offices multiple times a week, especially in the first few weeks of treatment, to setup their treatment plans and calibrate medication dosages [28]. These requirements, while important to prevent relapse, exacerbate the transportation burdens many individuals face as a result of limited reliable vehicle access or required travel time through public transit [29, 30]. Transportation barriers are especially common among rural populations, where fewer than 40% of MOUD providers are within walking distance of any form of public transportation and rural residents may spend five times longer traveling to a medical appointment compared to urban residents [29, 31].

Efforts to address transportation challenges for people with OUD are poorly documented and therefore not well understood. In this study, we explore this critical knowledge gap by documenting the costs and reach of a transportation assistance program (TAP) for individuals with OUD that was implemented as part of the HEALing (Helping to End Addiction Long-term^®^) Communities Study (HCS). HCS was a multi-site study in four states (KY, OH, NY and MA) focused on implementing evidence-based practices (EBPs) to significantly reduce opioid-related overdose fatalities in 67 urban and rural counties [32]. The overall study design was operationalized as a parallel-group, cluster randomized wait-list controlled trial where counties were randomized and divided into active intervention (Wave 1) or the wait-list control (Wave 2). Wave 2 counties received the intervention during the latter part of the study [32].

As part of this study, to address barriers to the uptake of and retention in MOUD treatment, EBPs were implemented to improve delivery of OUD services across counties. Because lack of access to affordable and reliable transportation was one barrier frequently reported, HCS KY designed and implemented a TAP to improve transportation options [27]. This paper first describes the HCS KY TAP and then provides results of the cost analysis of this program and the number of individuals impacted through measures of program reach.

## Description of program development and design

### Determination of transportation needs and community resources

The HCS KY TAP program was first designed and implemented during Wave 1 of the study. During this initial implementation, several “best practices” were identified and codified into the process for the Wave 2 counties. The implementation of the HCS KY TAP within MOUD partner agencies was led by HCS KY implementation facilitators. Partner agencies primarily consisted of opioid treatment programs or office-based treatment facilities within each county. Implementation facilitators conducted small group interviews with each partner agency to determine the specific obstacles faced by the populations served, including challenges to access and retention in MOUD treatment programs. Following recommendations from these interviews, thirty partner agencies agreed to receive transportation barrier relief services through the HCS KY TAP [33].

The HCS KY team explored various methods to improve client transportation access. During Wave 1 implementation, it was identified that public transportation and Medicaid transportation services should be surveyed as part of a broader landscape analysis prior to the HCS intervention implementation, the results of which shaped and affected which forms of transportation services were available for each agency. Additionally, this landscape analysis helped to establish contacts and standard operating procedures for purchasing public transportation passes or scheduling ride services. The HCS KY team also identified churches, local taxi services, and third-party ride service transportation vendors as potential options for local agencies. The HCS KY team prioritized pre-paid public transportation passes, fuel, and Uber gift cards to partner organizations due to difficulties in the payment process when working with local taxi services and ride service vendors.

Based on the interview findings and available local resources, which differed considerably among communities and required creativity and flexibility for meeting the local needs, a tiered funding model was developed for partner agencies. This model considered the number of clients served by each organization as a primary factor in determining financial allocation, with larger patient volumes considered reflective of greater transportation needs. Organizations were eligible for up to $1,000 per month if fewer than 100 MOUD patients were seen, $2,000 per month for 101–200 patients, and so on. These tiers were used for agencies in both rural and urban locations.

The assigned implementation facilitator arranged meetings to discuss these services with partner agencies. Once an agency accepted the offer of transportation assistance, HCS KY established contracts with those agencies and relevant third parties to provide the agreed upon mix of transportation options. Implementation facilitators worked with organizations to design internal client protocols and organizational support for working with the HCS KY team. The HCS KY TAP team managed the logistics of organizing the purchase, delivery, data collection, and troubleshooting of transportation programs.

### Transportation program design and administration

**Fig. 1:**
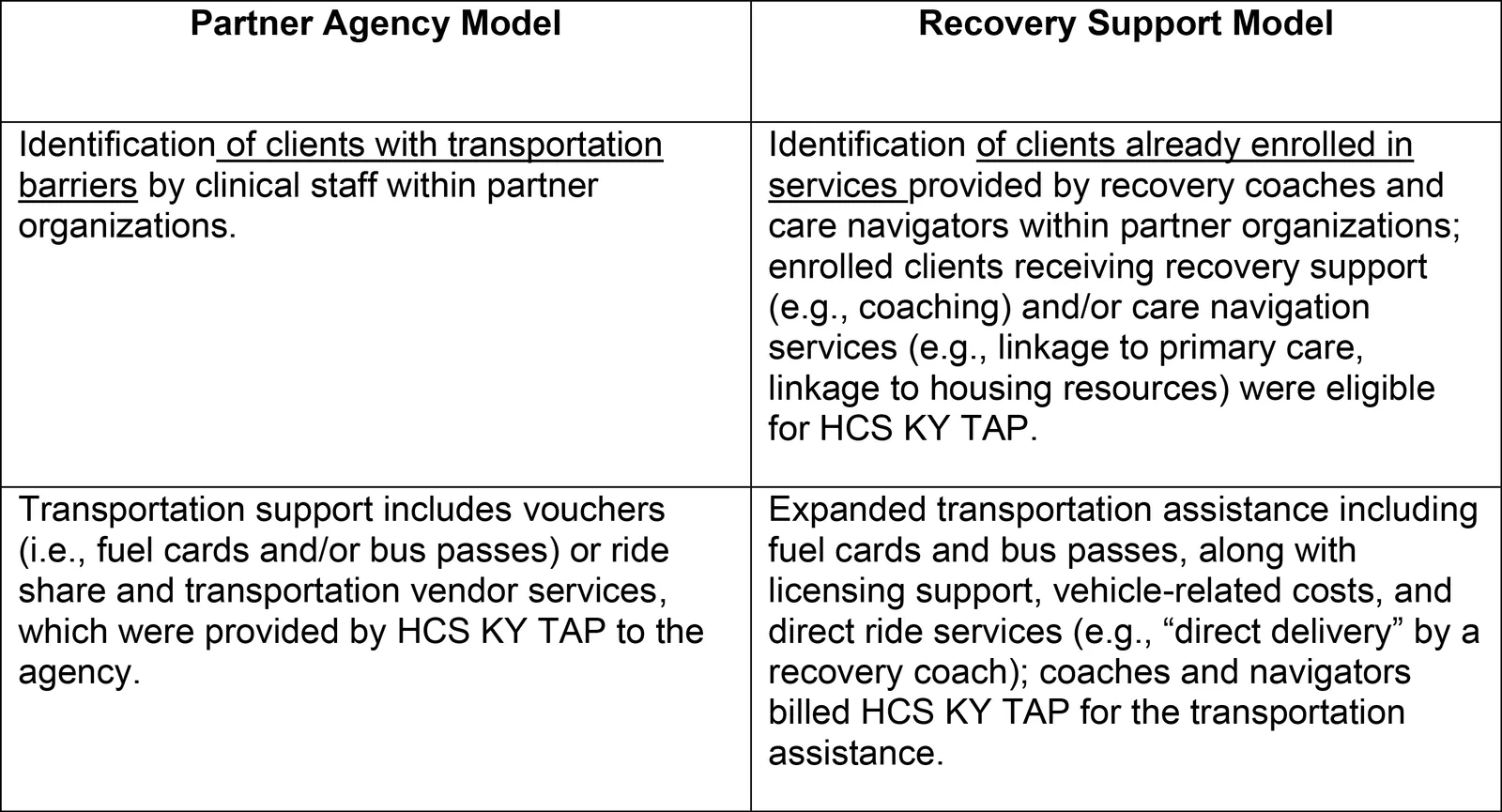
HCS KY TAP model differences

The HCS KY TAP used two mechanisms to provide funding for individuals in need. First, fuel cards, bus passes, rideshare reimbursement, and/or funds were issued to a partner agency to provide transportation directly to their clients (“partner agency model”). Second, recovery coaches from Voices of Hope – Lexington (VOH), trained individuals with lived experience, and care navigators from Bluegrass Care Navigators (BCN), certified social workers or nurses, who were placed at partner agencies through HCS KY, were able to request transportation-related funds to cover expenses that would support individuals in accessing or continuing treatment (“recovery support model”). The basic differences between these two models are displayed in Fig. 1.

In the partner agency model, clinical staff working in partner organizations identified individuals experiencing transportation barriers and offered them support. Clients receiving MOUD retention services or initiating treatment (e.g., first appointment) at any of the partner agencies in the eight HCS KY counties were eligible to receive HCS KY TAP services. There were no client income limitations in place for transportation assistance requests. Fuel cards were provided for individuals with a vehicle and for those without, solutions included public transportation passes (bus passes), transportation service financing (transportation service vendor invoicing and funding for vehicles leased by agency partners), and rides arranged by partner agencies (e.g., Uber gift cards).

For partner agencies, data collection and governance methods were employed to track HCS KY TAP resource use, including demographic breakdowns and service types provided. A Transportation Request Tracker was implemented using Smartsheet™ to collect data submitted by agencies for replenishments or reimbursement for transportation services provided to their clients and the cost of those services. This tracker collected data on the type of assistance being requested by the agency, quantity of cards/passes (if applicable), the request submission date, and whether it was approved by the HCS KY Team. These data served two functions: collecting data on the cost of the TAP and reporting to the university regarding the use and management of grant funds.

In the recovery support model, recovery coaches and care navigators implementing barrier relief services across study counties could access funding that addressed patients’ transportation needs as well as other social needs. Recovery coaches and care navigators were embedded in organizations to provide patient recovery support and targeted assistance for those most in need of retention services. Transportation support included bus passes and fuel cards like the partner agency model, but also included funds for acquiring driver’s licenses, car title expenses, purchasing non-motorized vehicles, and a new ride service program, where clients could ride with recovery coaches to and from MOUD appointments, referred to as direct delivery [34]. To be eligible to receive assistance through the HCS KY TAP, clients had to be 18 years of age or older, enroll in recovery coaching and/or care navigation, and receive MOUD in one of the eight HCS KY counties or from an approved Opioid Treatment Provider in a nearby county. In an effort to inform recovery coach and care navigator partner service delivery, clients were asked to identify barriers to care. Among those barriers, transportation was most commonly cited.

Although there is recognition that lack of transportation poses a significant barrier to treatment access and retention, few studies have implemented transportation assistance across a range of agencies and communities, so little is known about the costs of such services. Furthermore, examinations of costs in rural versus urban communities are rare. This dearth of data on costs poses a challenge to programs considering whether providing transportation assistance is economically feasible. This paper describes the results of the cost analysis of this multi-county transportation assistance program and the number of individuals reached, which can aid decision-makers in future efforts to reduce transportation barriers.

## Methods

### Data collection

This study uses data from eight HCS KY counties (five urban, three rural) in which the HCS KY TAP was implemented during Wave 2 of the study. Data on services and expenditures were collected during the HCS EBP implementation period from January 2023 through December 2023 (12 months). The HCS KY team used REDCap tools hosted at the University of Kentucky to collect data on the unique number of individuals reached by the HCS KY TAP across the eight counties [33, 35, 36]. Data points collected included total reach of individuals served and aggregated demographics (i.e., age, race/ethnicity, and sex).

In a separate REDCap instrument, partner agencies submitted a form each month to document transportation assistance that was provided to their clients (i.e., a bus pass, fuel card, Uber/Lyft ride, taxi, Uber/Lyft gift card, or other). Data regarding the number of individuals served through the transportation assistance partnerships, when available, included a de-identified unique client ID, the type of assistance and the date it was provided, the client’s county of residence, the initials of the agency staff who completed the form, and client demographic information (age, race, ethnicity, and sex). In some cases, due to staffing or agency data tracking limitations, it was not feasible for the partner agency to report person-level data on the transportation assistance they were providing. For these cases, HCS KY received aggregate counts from the agency of the number of individuals served each month while the strategy was active. The aggregated data were then organized by partner agency, county, month, and strategy.

### Measures

Across the HCS, reach was defined using an adaptation of the RE-AIM/PRISM framework as being the number of individuals who participated in an intervention [37–39]. For this work, reach was defined as the number of unique individuals receiving HCS KY TAP services through 30 partner agencies and the BCN/VOH teams serving the study counties; these counts were aggregated to the county-level. This measure was also used to calculate “average cost per individual reached” as a cost summary statistic. RE-AIM also characterizes reach in proportional terms; to measure the proportion reached by TAP services, we estimated a county-level measure of MOUD using data obtained from the Rapid Actionable Data for Opioid Response in Kentucky (RADOR-KY) system [40]. County-level MOUD in 2023 was calculated by summing county residents receiving treatment with buprenorphine from the prescription drug monitoring system along with Medicaid members receiving methadone and naltrexone for each county. While this measure is an undercount of individuals receiving MOUD because it does not capture methadone or naltrexone paid via private insurance or self-pay, it provides context for the other study variables. This county-level measure was used as the denominator to calculate the proportion of county-level MOUD patients reached by HCS KY TAP.

Transportation costs were captured in three categories: (1) vouchers, which included fuel cards, bus passes, and uber gift cards; (2) services, such as invoiced ride share, personal vehicle expenses and transportation services coordinated through the partner agency; and (3) direct delivery of transportation provided by recovery coach partners which was made up of driver time and mileage expenses. Costs during the study period were summed at the county level. To support comparisons of reach and costs between rural and urban counties, county designations from the 2013 National Center for Health Statistics Urban-Rural classification scheme for counties were used [41, 42].

### Data analysis

The cost analysis is framed from the community’s perspective, (i.e., a county), which represents multiple agencies engaging with and supporting the TAP. The goal is to highlight the resources that would be needed in any community planning to incorporate transportation assistance services.

To evaluate cost and reach, we used descriptive statistics for data collected from these agencies to understand the populations served, transportation services provided, the method of payment or reimbursement, and new services adopted with funding support from the HCS KY. Using the reach data obtained from the partner agency model and the recovery support model, we analyzed the counts by sex, race/ethnicity and age for each urban and rural county.

Summary cost statistics were calculated for total cost per county, costs segmented by rural - urban counties, and total costs across all transportation services delivered. Cost percentage per county, and urban-rural totals, were calculated to display how costs were distributed across counties. The study reach percentage was a calculation of the number of unique individuals reached in each county compared to the overall total. Lastly, the MOUD reach percentage divides the number of individuals reached by the county MOUD count obtained through the RADOR data to give an estimate of the proportion on MOUD who were impacted. To preserve anonymity, counties are identified in this manuscript by their urban or rural status (U or R) and a number identifier.

Reach data were exported and analyzed at the county-level using SAS v9.4 (Cary, NC). This study protocol (Pro00038088) was approved by Advarra Inc., the HEALing Communities Study Single Institutional Review Board (ClinicalTrials.gov Identifier: NCT04111939).

## Results

A total of 7,923 unique individuals received HCS KY TAP services through the partner agency model and recovery support model during the implementation period of January through December of 2023, as shown in Table 1. Most individuals reached by these services were white (81.8%; *n*=6,483), female (51.9%; *n*=4,112) and between the ages of 35-54 years old (59.8%; *n*=4,737). Over three-fourths of unique individuals reached through the TAP (75.7%; *n*=5,999) were in the five urban counties.

**Table 1:** Transportation strategy reach by county and demographics

Individuals Reached		*Rural*			Rural Total	*Urban*					Urban Total	*Overall Total*	*%*
		R1	R2	R3		U1	U2	U3	U4	U5			
	Total	1198	502	224	1924	782	291	277	3561	1088	5999	7923	
**Demographics**													
Sex													
	Male	486	254	115	855	378	134	126	1657	501	2796	3651	46.1%
	Female	708	215	109	1032	402	157	151	1793	577	3080	4112	51.9%
	Other	2	1	0	3	1	0	0	7	0	8	11	0.1%
	No response	2	32	0	34	1	0	0	104	10	115	149	1.9%
Race/Ethnicity													
	Hispanic	2	2	3	7	5	3	0	37	6	51	58	0.7%
	Black	5	1	0	6	28	13	0	342	43	426	432	5.5%
	White	871	462	221	1554	747	270	277	2664	971	4929	6483	81.8%
	Non-Hispanic_Other	2	5	0	7	1	4	0	34	5	44	51	0.6%
	No response	318	32	0	350	1	1	0	484	63	549	899	11.3%
Age													
	<18	1	0	0	1	0	0	0	0	0	0	1	0.0%
	18-34	440	134	58	632	123	80	88	1016	243	1550	2182	27.5%
	35-54	671	254	151	1076	531	185	149	2051	745	3661	4737	59.8%
	55+	86	80	15	181	127	26	39	390	92	674	855	10.8%
	No response	0	34	0	34	1	0	1	104	8	114	148	1.9%

Note: Reach data reflects 257 of 298 (86.2%) agency-months of data

Costs for TAP totaled $437,481.69 for all services provided over the course of 2023. Expenses were largest among the urban counties (73%; $318,317.15), which averaged $53.06 per person. Within the urban counties, we worked with 23 agency partners, and the average agency costs were $10,993.72. Across the three rural counties, costs averaged $61.94 per person reached (1,924) and accounted for 27% of expenses ($119,164.54). Among the seven agency partners across the three rural counties, average agency costs were $9,235.38. The majority of transportation service costs were for voucher expenses, such as fuel cards, Uber cards, and bus passes (40.3%; $176,310.50), which were largely implemented within urban counties ($143,550.50). This was followed by invoices for services, which included ride share invoices, leased partner agency vehicle expenses, and transportation agencies (32.3%; $141,192.61) and costs for direct delivery services (27.4%, $119,978.58). The average cost per individual reached across both urban and rural counties was $55.22 (Table 2).

**Table 2:** Transportation strategy cost by county and county type

County Type	Vouchers*	Services**	# of Agencies	Average Agency Cost	Direct Delivery***	Total Cost Per County	Cost %	Individuals Reached	Study Reach	County MOUD****	MOUD Reach %	Average Cost Per Individual Reached
**Urban**												
U1	$32,640.00	$2,632.67			$20,379.23	$55,651.90	13%	782	10%	554	141%	$71.17
U2	$21,978.00	$-			$2,641.97	$24,619.97	6%	291	4%	1063	27%	$84.60
U3	$11,360.00	$-			$510.51	$11,870.51	3%	277	3%	1133	24%	$42.85
U4	$70,972.50	$93,045.78			$32,219.32	$196,237.60	45%	3561	45%	8276	43%	$55.11
U5	$6,600.00	$13,626.50			$9,710.67	$29,937.17	7%	1088	14%	1205	90%	$27.52
** Urban Total**	$143,550.50	$109,304.95	23	$10,993.72	$65,461.70	$3,18,317.15	73%	5999	76%	12,231	49%	$53.06
**Rural**												
R1	$11,860.00	$24,835.66			$26,518.78	$63,214.44	14%	1198	15%	876	137%	$52.77
R2	$13,900.00	$7,052.00			$10,374.18	$31,326.18	7%	502	6%	973	52%	$62.40
R3	$7,000.00	$-			$17,623.92	$24,623.92	6%	224	3%	277	81%	$109.93
** Rural Total**	$32,760.00	$31,887.66	7	$9,235.38	$54,516.88	$119,164.54	27%	1924	24%	2126	90%	$61.94
**Overall Total**	$176,310.50	$141,192.61	30	$10,583.44	$119,978.58	$437,481.69	100%	7923	100%	14,357	55%	$55.22

‘-‘ = no resources expended

* Vouchers = Uber cards, fuel cards and bus passes given to individuals identified by partner agencies

** Services = Ride share, vehicle expenses, and transportation services purchased by partner agencies and then invoiced to the University

*** Direct delivery = This is not attributed to an agency and was provided by our VOH & BCN partners. Drivers’ time, mileage expenses, and other transportation services provided directly by Recovery Coaches

**** County MOUD = Residents receiving buprenorphine within that county and Medicaid members receiving Methadone and Naltrexone

The unique number of individuals reached in urban counties was 76% of the total reach, while urban counties only accounted for 73% of the expenses. The average cost per individual reached was lower for those in urban counties ($53.06) compared to those in rural counties ($61.94), as shown in Table 2.

## Discussion

Considerable resources were invested in transportation services for HCS KY communities reflecting the true barrier that transportation represents for many individuals seeking MOUD. These direct investments in transportation were intended to improve retention in care for those receiving MOUD by preventing drop-out due to transportation barriers. Through this program, nearly 8,000 unique individuals were reached across the 8 study counties. Urban counties reached many more individuals than rural counties, which likely reflects variation in population size. Of note, the Kentucky Association of Counties (KaCo) 2023 population estimates show a 94% to 6% urban-rural split for the eight study counties [43]. Therefore, while rural counties represent just 6% of the population, 27% of individuals reached through the TAP lived in rural counties. This speaks to the vast need for transportation services in rural counties, and through intentional effort, HCS KY TAP was able to reach a disproportionate number of rural people. At the same time, we also acknowledge that our largest county, which accounted for 75% of the total population, only represented about 45% of the total individuals reached, suggesting we were likely under-scaled in that county despite efforts to engage a considerable number of partner agencies. At the same time, the nearly 8,000 individuals reached represent a substantial percentage of county residents receiving MOUD. By leveraging data from the RADOR-KY [40], we estimate that HCS KY TAP may have reached approximately half of county residents receiving MOUD in 2023; however, it should be noted that our county MOUD measure does not include self-pay and privately insured individuals receiving methadone or naltrexone. It should also be noted that two of the counties show a reach of greater than 100%, which may be explained by the TAP design in which the counts reflect where people received MOUD, not where they resided; RADOR-KY’s MOUD data reflects county of residence. It may be that in some cases individuals lived within one county but received MOUD in an adjacent county (i.e., a study county). In these scenarios, the individual would be included in the TAP reach count, but in RADOR-KY, they would not be reflected because they lived in a non-study county. Despite these limitations, the RADOR-KY data provide some contextualization of the magnitude of reach achieved by HCS KY TAP.

Average cost per individual varied significantly by county type (urban/rural) with costs ranging from $27.52 (U5) to $109.93 (R3) per person reached. Results also identified wide variations within county types, which highlights the heterogeneity of need and available service options between counties. As shown on Table 2, the dominant type of transportation service available and used varied by county, with urban counties relying more on vouchers, such as bus passes and fuel cards, and ride-share services. In contrast, rural counties used more direct delivery approaches which were more costly per individual, suggesting the need to customize transportation programs to fit the local environment. Additionally, since we largely relied on partner agencies to implement these programs, the voucher program was likely most popular due to the limited resources required by the agency when compared to coordinating transportation services, covering up front costs and then invoicing for reimbursement of those expenses.

The volume and cost of TAP services provided varied widely among the differing agencies across and within each county with many counties only having one or two agencies utilizing the bulk of services. Since we did not work with every agency in each county and because agency variability may have stemmed from agency resource limitations, and not community need, we only summarized the average cost per agency across the urban/rural groupings to prevent agency identification. This limited our ability to determine unique agency differences, which may have led to their cost differences.

One key finding from this study was the need to be both creative and flexible when identifying transportation resources across communities, as there cannot be a one-size-fits-all approach. Additionally, this study highlights a variety of barriers experienced during implementation which should be considered in future programs. Common local barriers identified during the HCS KY study included: lack of reliable vehicles (e.g., vehicle repair needs), limited public transportation, and partner agency resource limitations for supporting transportation programs. Even when public transportation was available within a county, patients still faced barriers in terms of scheduling challenges or living a considerable distance from the nearest bus stop. Other barriers included the lack of economical ride service vendors, high fuel prices, and clients’ household disqualification from Medicaid transport. Medicaid limitations were especially important during the study period as vehicle ownership (even if the vehicle was inoperable) led to automatic disqualification for non-emergency medical transportation (NEMT) programs until July 2024 [27, 44]. In general, the partner MOUD agencies lacked dedicated resources for transportation assistance before HCS KY TAP, with the exception of small grants and two vans operated by two treatment centers.

This study has several limitations. First, implementation of these programs required targeted staff resources, as each study county presented with differing obstacles. Agency partners encountered challenges with transportation assistance distribution, which may have limited the number of individuals who received services and the related expenses. Challenges included having insufficient staff time to identify patients with transportation needs, build contractual agreements, and participate in ongoing facilitation (e.g., meetings between implementation facilitators and agencies). These costs were not captured within this study and are therefore not reported but are real costs that would need to be considered before replicating this work. In addition, since agencies were required to submit demographic data on patients receiving HCS KY TAP assistance before receiving more transportation resources, those who did not provide the required data were unable to utilize the total funding available for transportation support. Additionally, staff turnover within each agency was often cited as a reason why services were not fully implemented or when data was lost or not collected. In situations where staff left their positions before a replacement was hired, there was no opportunity for cross-training or a hand-off of transportation support responsibilities. Therefore, data were limited by the availability of agency staff to collect and provide data to the research team. Based on timelines of partner agency implementation, 30 agencies implemented HCS KY TAP for a total of 298 agency-months, and data was received for 257 of those agency-months (86.2%). Since the transportation tracking process required a separate system for each agency, housed outside of their EMR, management of this program required staff time to document support and manually pull reports each month, which may have led to data retrieval errors. Turnover among recovery coaches and care navigators deployed in partner agencies also limited access to the transportation support provided until new staff were hired. In follow-up meetings between the HCS KY team and partner agency staff, this turnover and the added workload was mentioned as a reason transportation support was not being provided or data was missing.

Other limitations of the study include our inability to calculate how costs differ across transportation services per unique individual, determine the total number of trips taken as part of the TAP program, and identify a true proportion of people reached across the full eligible population. Since the majority of services provided within the TAP were vouchers (i.e. bus passes and fuel cards) and our study did not consent TAP recipients to participate in data collection, we were limited to receiving agency reports of vouchers issued. This limitation meant we were unable to determine how many MOUD visits the vouchers covered for each unique individual, and we were unable directly test the association between transportation assistance and retention. Future research, which consents and tracks unique individuals using transportation support services and measures treatment retention is much needed to expand upon this work. Additionally, a single individual may have received assistance from both the partner agency model (e.g., received a voucher) and the recovery support model; these two models tracked reach separately. Finally, we were unable to determine over the 12-month implementation period how many patients had a transportation need in each agency or county since all patients from all MOUD providers were not surveyed. However, using the data we have available, a county MOUD utilization calculation was used to provide some contextualization of the reach of the TAP.

Although transportation challenges are often framed within the context of social determinants of health (SDOH), which include economic stability, access to education and healthcare, neighborhood and built environment, and social and community context, they represent just one piece of a larger set of obstacles to care [23, 45]. Transportation needs overlap with several SDOH domains (e.g., access to care, infrastructure/built environment, social/community context) but addressing it effectively and sustainably requires a wider lens [19–22, 27]. Telehealth has emerged as an important modality for delivering all types of healthcare, including treatment for OUD, where research has already identified improvements in treatment access and retention [46, 47]. Among these partner agencies, telehealth had greatly expanded during the COVID-19 pandemic, and in 2023, most still offered telehealth but also continued to provide in-person services, which is why lack of transportation was still considered a major barrier to care [27]. As this trend of increased telehealth continues, it will be interesting to explore the impact it may have on the need for transportation support services.

## Conclusion

This analysis of the costs and reach of the HCS KY TAP supports the significance of transportation as a barrier to MOUD treatment, which is broadly experienced by individuals in both urban and rural settings, and provides new knowledge on the cost and reach of a program which was implemented to address this barrier. Transportation support is a critical area for investment to promote improved OUD treatment service delivery and to address SDOH in terms of access, infrastructure, and the environmental context among vulnerable population subgroups. Our findings demonstrate that targeted transportation support services are feasible and may effectively support the recovery journey of this vulnerable population. By building partnerships with local and regional service providers, programs can flexibly address treatment barriers and allocate resources to reach individuals seeking treatment. Research is needed to directly examine the associations between transportation assistance and the likelihood of being retained on MOUD for at least six months, which has been proposed as the minimum effective treatment duration [48]. Further study is also needed to determine which types of transportation assistance are most beneficial in rural areas and why average per person costs were so variable among counties. Finally, the changes made to Medicaid transportation policies to clarify household working vehicle limitations which ensured all household members have transportation options were a valuable policy change for individuals needing these services and the impact of future policies that may allow reimbursements to healthcare providers delivering patient transportation services should be explored.

## Data Availability

Organization level data reported in the current study are not publicly available to protect the privacy of organizational partners.
